# Study of Ag Nanoparticles in a Polyacrylamide Hydrogel Dosimeters by Optical Technique

**DOI:** 10.3390/gels8040222

**Published:** 2022-04-05

**Authors:** Yasser S. Soliman, Soad M. Tadros, Wafaa B. Beshir, Gamal R. Saad, Salvatore Gallo, Laila I. Ali, Magdi M. Naoum

**Affiliations:** 1 National Center for Radiation Research and Technology (NCRRT), Egyptian Atomic Energy Authority, Cairo 11787, Egypt; yasser_shabaan@hotmail.com (Y.S.S.); dr.wafaa@live.com (W.B.B.); 2 Chemistry Department, Faculty of Education, Ain Shams University, Cairo 11566, Egypt; lailaali@edu.asu.edu.eg; 3 Chemistry Department, Faculty of Science, Cairo University, Cairo 12613, Egypt; grsaad@sci.cu.edu.eg (G.R.S.); magdinaoum@yahoo.co.uk (M.M.N.); 4 Physics Department, “Aldo Pontremoli” Milano University, 20133 Milan, Italy; salvatore.gallo@unimi.it

**Keywords:** dosimetry, hydrogel dosimeter, Ag nanoparticles, surface plasmon resonance band, absorption spectroscopy, gamma radiation

## Abstract

The dosimetric characteristics of hydrogel dosimeters based on polyacrylamide (PAC) as a capping agent incorporating silver nitrate as a radiation-sensitive material are investigated using UV-Vis spectrophotometry within the dose range 0–100 Gy. Glycerol was used in the hydrogel matrix to promote the dosimetric response and increase the radiation sensitivity. Upon exposing the PAC hydrogel to γ-ray, it exhibits a Surface Plasmon Resonance (SPR) band at 453 nm, and its intensity increases linearly with absorbed doses up to 100 Gy. The results are compared with the silver nitrate gel dosimeter. Glycerol of 15% in the hydrogel matrix enhances the radiation sensitivity by about 30%. PAC hydrogel dosimeter can be considered a near water equivalent material in the 400 keV–20 MeV photon energy range. At doses less than 15 Gy, the PAC hydrogel dosimeter retains higher radiation sensitivity than the gel dosimeter. The total uncertainty (2σ) of the dose estimated using this hydrogel is about 4%. These results may support the validity of using this hydrogel as a dosimeter to verify radiotherapy techniques and dose monitoring during blood irradiation.

## 1. Introduction

Gel dosimeters can measure radiation dose distributions in three dimensions (3D), making them excellent dosimetric tools for external beam radiotherapy. These dosimeters are important for dose verifications of radiotherapy and the establishment of treatment planning systems as they have a good spatial resolution property [1,2]. Hydrogels are nearly tissue equivalent and can be molded to any desired shape or form [3,4] There are different classes of gel dosimeters, particularly the Fricke gel and polymer gels [4]. Fricke gel (FG) systems are based mainly on ferrous ions (Fe^2+^) in a gel matrix. The interaction of ionizing radiation with the molecules of the hydrogel, and the consequent free-radicals formation, activate different chemical routes that lead to the oxidation of ferrous ions (Fe^2+^) to ferric ions (Fe^3+^) with oxidation yield proportional to the absorbed dose. This variation is detectable by Nuclear Magnetic Resonance (NMR) and Magnetic Resonance Imaging (MRI) [5,6]. Furthermore, adding a suitable metallic-ion indicator to the FG dosimeters makes these systems capable of being analyzed by optical techniques [7,8,9,10,11]. These gels are simple to prepare and less expensive, and different gel matrices such as gelatin, agarose, and poly(vinyl-alcohol) (PVA) were largely studied [3,4,7,12,13,14]. The major drawback of FG dosimeters is the diffusion of ferrous and ferric ions, which leads to a gradual blurring of the dose pattern with time after irradiation. [15,16,17]. The diffusion limitation of the FG can be overcome by using a different radiochromic gel called leucocrystal violet micelle gel (LCV gel) [17,18].

Researchers are currently working on improving gel dosimeter sensitivity and efficiency and finding new dosimeters of suitable properties for radiation technology applications. Nanoparticles such as gold, bismuth, platinum, and silver can be incorporated into the gel to enhance the sensitivity and efficiency for a lower dose range [19,20,21]. Moreover, radiation-induced AgNPs in silver nitrate solutions could be successfully applied for radiation detection and dosimetry [22,23] and dose enhancement in radiotherapy applications [19,24,25,26]. AgNPs are an exciting alternative for dose enhancement in radiation therapy due to their inexpensive cost compared to gold nanoparticles [25] and antitumor activity [27,28]. The silver nitrate dosimeter features a linear response and good measurements reproducibility [22]. The influence of γ-rays on AgNO_3_ solution had been examined [29,30,31,32,33,34]. A liquid detector based on silver nitrate and 1% sodium citrate is introduced, where the ionizing radiation induces the formation of spherical AgNPs as recognized by the appearance of a sharp peak around 410 nm in the absorbance spectrum of the colloidal solution [35]. However, this study did not address most of the dosimetric characteristics [35].

The radiation-sensitive silver nitrate was mixed with gelatin (8%) as a stabilizing agent to minimize the agglomeration of the formed AgNPs [23]. This gel exhibits an SPR band at 450 nm related to AgNPs formed upon irradiation. This dosimeter has a linear dose–response function up to 100 Gy. The increase in Ag^+^ ion concentrations in the gel considerably improves the radiation dose sensitivity. The overall uncertainty (2σ) of dose estimation in the range of 5–100 Gy was found to be ≈4.65%; thus, it can be applicable for radiotherapy dose measurements and blood irradiation. The response of irradiated AgNO_3_ gel displays good stability over a month after irradiation when kept at 6 °C. In contrast, it shows poor stability and considerable response growth when stored in the dark or light after irradiation. The gel response has a temperature coefficient of ~0.339% per 1 °C [23]. Recently, Tadros et al. [36] improved the sensitivity of this gel dosimeter by using isopropanol in the gel matrix and decreasing the gel content to 4% instead of the 8% previously used [23].

The present investigation aims to develop a radiochromic hydrogel dosimeter for silver nitrate using polyacrylamide (PAC), as a new capping agent for preventing aggregation of Ag [37,38,39,40], and to study the suitability of its use in low-dose dosimetry applications (radiotherapy and blood irradiation). The γ-rays induced AgNPs in the PAC hydrogel dosimeter, the effect of silver nitrate concentration, the glycerol content on the dosimeter response, and the dose–response functions were investigated using a UV-Vis spectrometric technique. The overall combined uncertainties (at 2σ) associated with the calibration and the energy dependence were calculated. In addition, the effects of temperature during irradiation on the response function and post-irradiation stability were described. Finally, we compared the results of silver nitrate hydrogel dosimeters with the previous results of silver nitrate gel dosimeters [23,36].

## 2. Results and Discussion

### 2.1. Absorption Spectra of PAC Hydrogel Dosimeter

**Figure 1:**
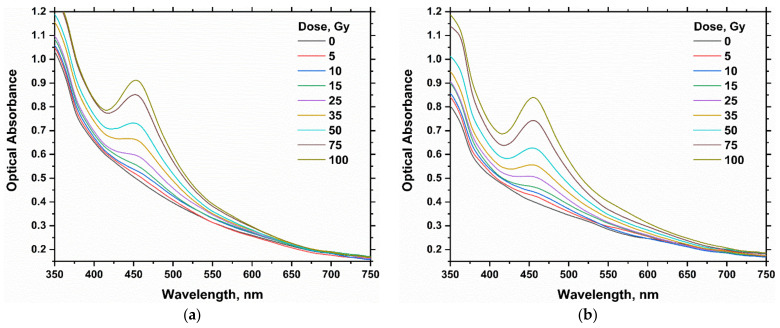
Optical absorption spectra of PAC hydrogel dosimeter unirradiated and irradiated at various absorbed dose values up to 100 Gy for PAC dosimeter with 100 mM AgNO_3_ (**a**) and PAC dosimeter with 150 mM AgNO_3_ (**b**).

The optical absorption spectra of silver nitrate PAC dosimeter (100 and 150 mM) in the 0–100 Gy dose range are shown in Figure 1a,b. The unirradiated AgNO_3_ gel is colorless and does not have prominent peaks in the visible range. The color of the PAC hydrogel dosimeter turns into a visual yellow color with irradiation, as shown in Table 1. The intensity of the yellow color and optical absorbance increases with the increase in the absorbed doses. A significant main band at 453 nm, corresponding to a band of SPR of AgNPs absorption [23], distinguishes this color. The band intensity increases progressively as the radiation dose increases, with no noticeable shifts in the band position. While, as the absorbed dose increases, the broadening of the peak gradually decreases.

Furthermore, the position of the SPR band is found to be mainly dependent on the size of the formed nanoparticles [41] and the nearby intermediate [42,43]. This indicates that γ-irradiation-induced nucleation of silver nanoparticles is dependent on the absorbed dose [44]. At higher doses around 100 Gy, there is no pronounced shift in the main absorption band with the dose, [41,45]. This suggests that at high irradiation doses below 100 Gy, the nucleation event is more than the total ions. On the other hand, at low doses where the nucleation event is less than the total ions, the radiation produced larger sizes of Ag following aggregation [44].

PAC has a dual function: it acts as a steric stabilizer, preventing agglomeration of gamma-induced AgNPs, and it can form complexes with Ag^+^ ions via interaction with the amino (–NH_2_) groups of PAC. This coordination effect can significantly slow the reduction to enable kinetic control [39]. The PAC polymer and AgNPs interact by charge transfer from the metal particles to the nitrogen sites on the polymer side chains, as shown by S Mukherjee and M Mukherjee [40].

### 2.2. The Effect of Silver Nitrate Concentration on PAC Hydrogel Dosimeter

**Figure 2:**
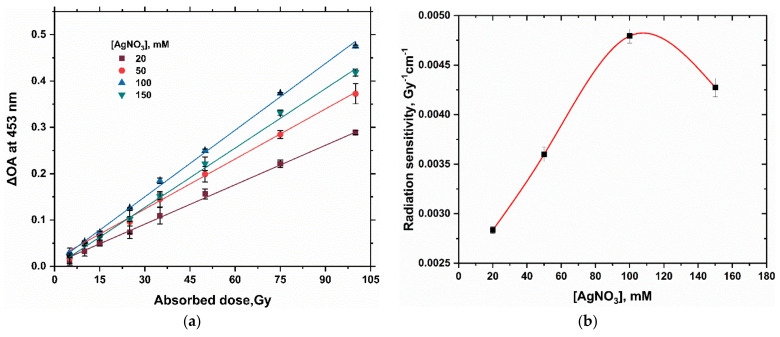
(**a**) illustrates the dose–response curve of silver nitrate PAC hydrogel dosimeter (2% PAC) at different AgNO_3_ concentrations; net absorbance change at 453 nm as a function of absorbed dose (5–100 Gy). (**b**) shows the radiation sensitivity of silver nitrate PAC dosimeter as a function of [AgNO_3_]. The solid red line is a guide for the eye obtained using a basis spline function. The error bars denote the standard deviation of the mean values.

The dose–response functions of PAC hydrogel dosimeter at various silver nitrate concentrations (20, 50, 100, and 150 mM AgNO_3_) are shown in Figure 2a. Table 2 shows the radiation sensitivity and the percent variation compared to the standard 20 mM Ag^+^ concentration. The dose responses are linear, with R^2^ values of 0.9992, 0.9975, 0.9987, and 0.9973 for silver nitrate concentrations of 20, 50, 100, and 150 mM, respectively.

The response of this dosimeter increases with increasing [Ag^+^] concentrations up to 100 mM, then the response decreases at 150 mM as investigated in the radiation sensitivity curve shown in Figure 2b. As the concentration of the Ag^+^ ions increased from 50 to 100 mM, the radiation sensitivity was found to be increased by approximately 33%. As a result, the appropriate composition should be selected based on the dose range required. The 100 mM Ag^+^ gel dosimeter can be used for doses ranging from 5.0 Gy to 100.0 Gy, as a useful range for blood irradiation and radiotherapy dosimetry.

### 2.3. The Effect of Glycerol on the Response of PAC Hydrogel Dosimeter

**Figure 3:**
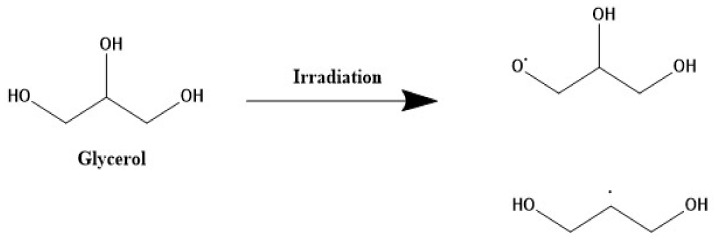
Represent the free radicals of Glycerol.

Glycerol is a non-toxic, non-volatile, and biodegradable material. In addition, it can dissolve a wide range of compounds that have poor solubility in water and be a promising candidate as a “green solvent” [46,47]. Thus, it was selected in the preparation of the hydrogel matrices. Recent works have also demonstrated that glycerol can be used as a solvent and reducing agent to generate metal nanoparticles [47,48]. Free radical species are generated for glycerol upon irradiation, as shown in Figure 3. The obtained glycerol radicals can serve as a reducing agent for silver ions [47,48]. In addition, irradiation of glycerol in an aqueous medium produces solvated electrons which serve as a reducing agent for Ag^+^. Glycerol yields solvated electrons greater than other alcohols [47].

**Figure 4:**
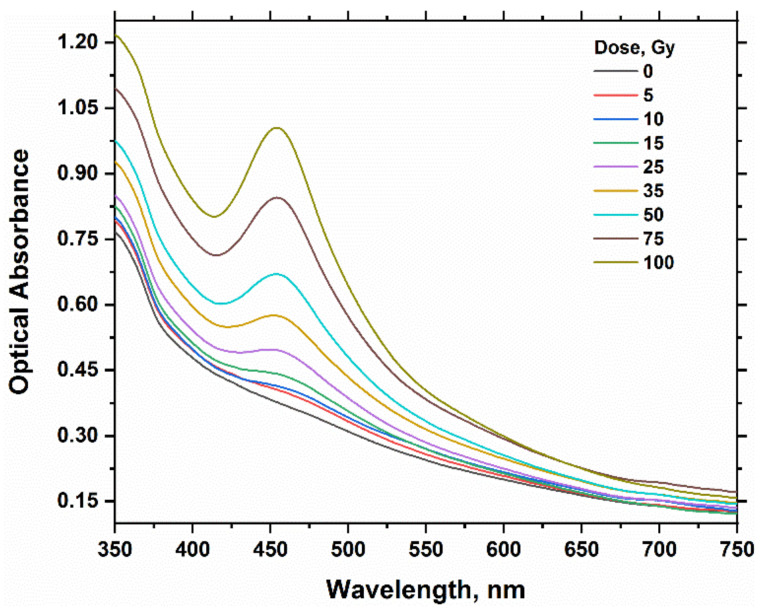
Optical absorption spectra of PAC hydrogel dosimeter (100 mM AgNO_3_ and 15% glycerol) unirradiated and irradiated at various absorbed doses values up to 100 Gy.

**Figure 5:**
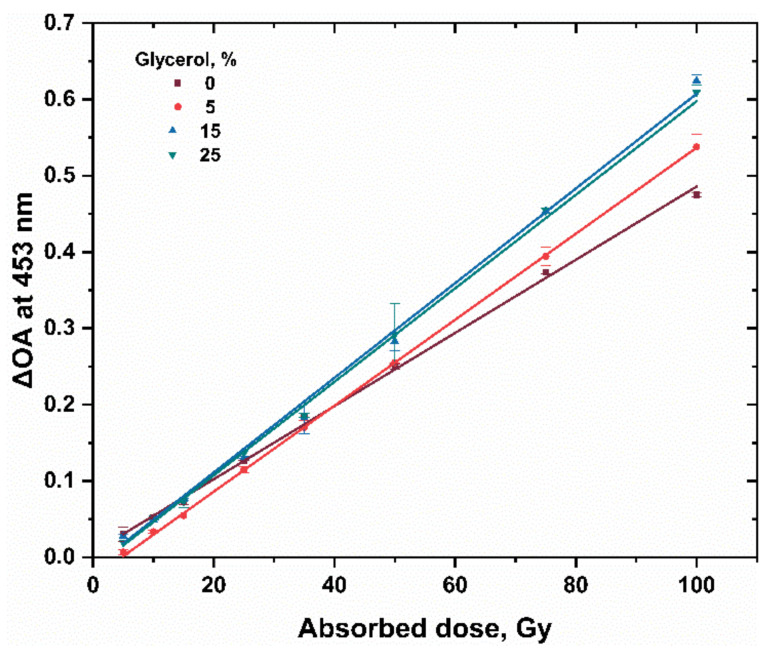
Dose–response curve of PAC hydrogel dosimeter (2% PAC) at different glycerol content, %; net absorbance change at 453 nm as a function of absorbed dose (5–100 Gy for 0, 5, 15, and 15% of Glycerol).

**Figure 6:**
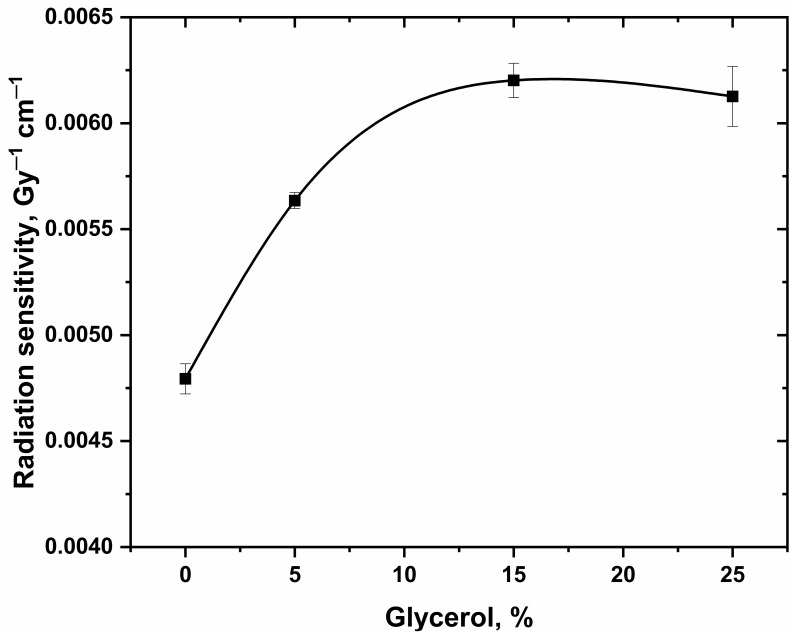
Radiation dose sensitivity of PAC hydrogel dosimeter as a function of glycerol concentration (%). The solid line is a guide for the eye obtained using a basis spline function. The error bars represent the one standard deviation of the mean values.

Figure 1 and Figure 4 represent the absorption spectra for the hydrogel dosimeters without and with 15% glycerol, respectively. The spectra of both types have nearly the same peak features, but the absorbance change is highly significant with the absorbed dose in the case of using glycerol in the hydrogel matrix. This result indicates also the reduction of Ag^+^ ions into Ag NPs as in the case of hydrogel dosimeter without glycerol. Figure 5 shows the dose–response curves for PAC hydrogel dosimeter at different content of glycerol %. The intensity of the absorption band increases linearly with increasing dose up to 100 Gy, with correlation coefficients (R^2^) of 0.9987, 0.9997, 0.9991, and 0.9968 for glycerol 0, 5, 15, and 25%, respectively, indicating the goodness of linearity. The hydrogel response increased as the glycerol content increased from 0 to 15%. This result indicates that glycerol significantly reduces the Ag^+^ ions in the hydrogel into AgNPs. The increase in glycerol content in the PAC matrix from 0 to 15% improves the radiation sensitivity by ≈30%, as shown in Figure 6 and Table 3. Then, the steady state is reached at 25% glycerol. Consequently, it is recommended to use 15% glycerol on the hydrogel matrix to increase the radiation sensitivities in the radiotherapy dose applications.

### 2.4. Effect of Irradiation Temperature on the Response of PAC Hydrogel Dosimeter

**Figure 7:**
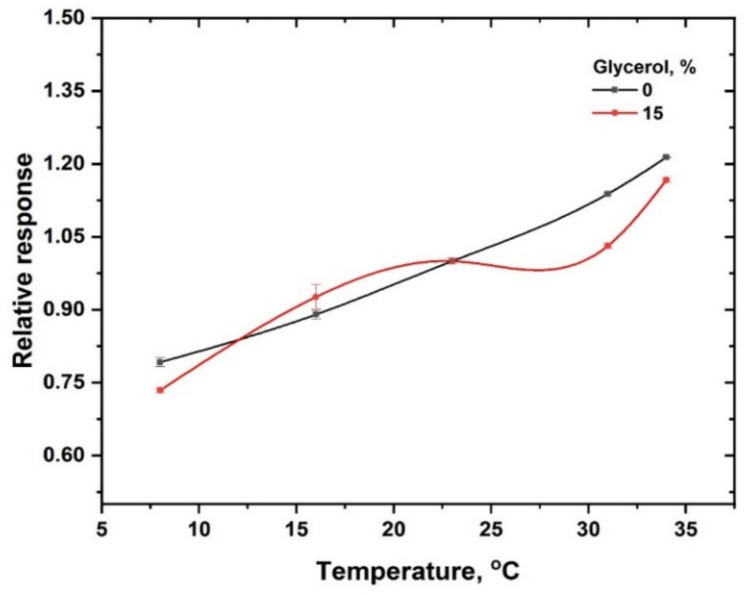
Variation of OA at 453 nm normalized to the value at room temperature as a function of irradiation temperature. The solid lines are a guide for the eye obtained using a basis spline function.

The effect of irradiation temperature on the PAC hydrogel dosimeter (100 mM) response in the range of 8–34 °C was investigated by irradiating the gel dosimeters with γ-rays to an absorbed dose of 50 Gy normalized to response value at 23 °C (see Figure 7). The results reveal that the response of the PAC hydrogel dosimeter increases linearly with irradiation temperature over the range of 8–34 °C. The temperature coefficient is +1.6 ± 0.9% per °C. In comparison, the temperature has a low impact for gel dosimeters containing 15% glycerol in the range of 16–31 °C; the temperature coefficient is +0.69 ± 0.1% per °C. For the silver nitrate PAC hydrogel dosimeter, it is strongly recommended to calibrate it under actual processing conditions, i.e., in-plant calibration, to minimize the errors resulting from temperature rise [49]. Alternatively, correction to temperature effect has to be applied.

### 2.5. Stability of Silver Nitrate PAC Hydrogel Dosimeter

**Figure 8:**
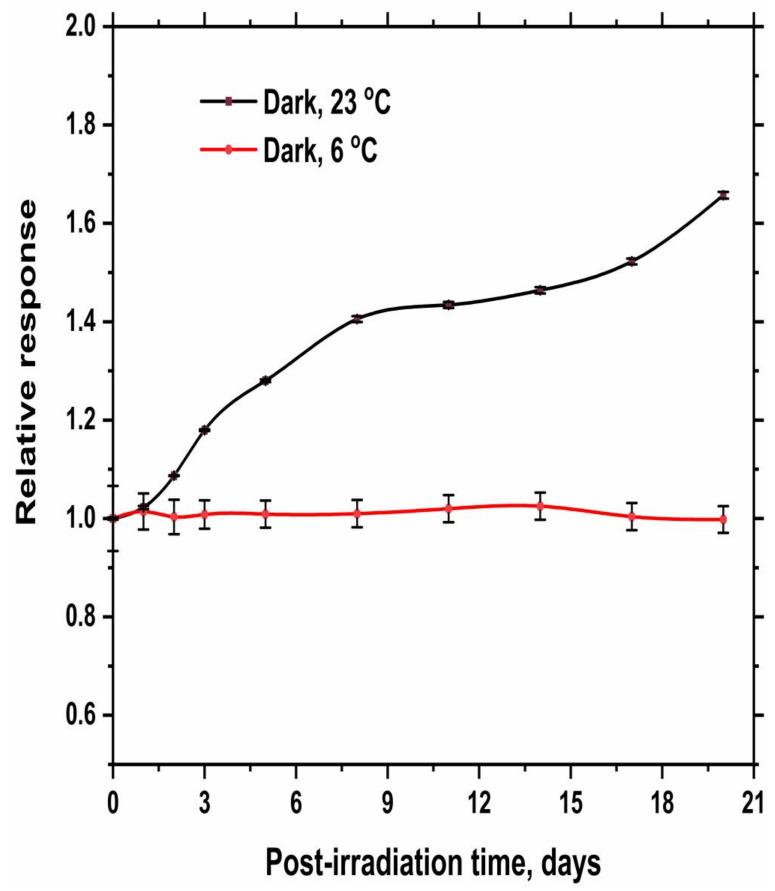
Relative response of irradiated hydrogel dosimeter (100 mM AgNO_3_ and 0% Glycerol) to the response measured immediately after irradiation (zero time) as a function of storage time. The hydrogel dosimeters were irradiated at 50 Gy and then stored in a dark place at different temperatures (6 °C and RT, 23 °C). The solid lines are a guide for the eye obtained using a basis spline function.

**Figure 9:**
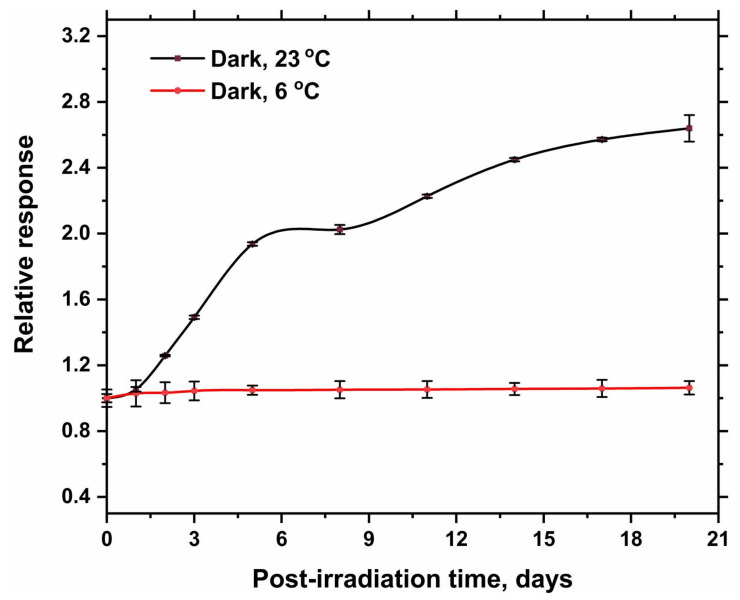
Relative response of irradiated hydrogel dosimeter (100 mM AgNO_3_ and 15% Glycerol) to the response measured immediately after irradiation (zero time) as a function of storage time (days). The hydrogel dosimeters were irradiated at 50 Gy and then stored in a dark place at different temperatures (6 °C and RT, 23 °C). The solid lines are a guide for the eye obtained using a basis spline function.

Figure 8 and Figure 9 illustrate the change of relative response of irradiated hydrogel dosimeter stored in a dark place at different temperatures (6 °C and RT, 23 °C) with time for the hydrogel of 0 and 15% glycerol, respectively. The results demonstrated that both irradiated hydrogel dosimeters stored at 6 °C are nearly stable over 15 days. Under room temperature, the response of both hydrogel dosimeters increased significantly with storage time [23]. However, the rate of increasing responses is higher in the case of using 15% glycerol than the hydrogel of 0% glycerol. Thus, it is recommended to store the dosimeter at 6 °C to minimize the continuous reduction of Ag^+^ ions with time.

### 2.6. Effective Atomic Numbers and Water Equivalency of PAC Hydrogel Dosimeter

Table 4 displays the composition and fraction of atoms by weight for the PAC hydrogel dosimeters without and with 15% glycerol, respectively. We used the X-COM program [50] to obtain the fraction by weight for these dosimeters.

**Figure 10:**
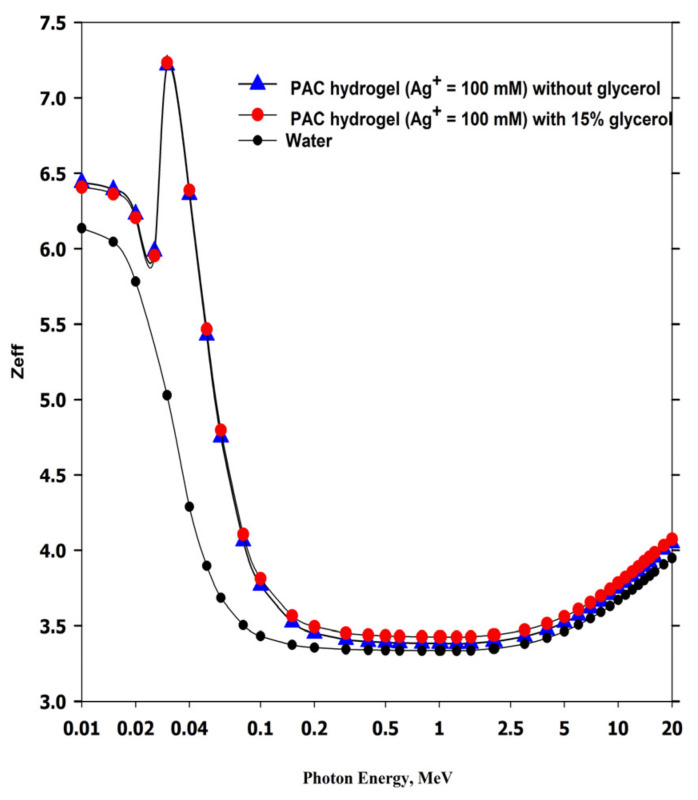
Effective atomic number (Z_eff_) of PAC hydrogel dosimeter (100 mM Ag^+^) without and with glycerol and compared with water as a reference material.

Effective atomic numbers (Z_eff_) at different energies were estimated using the Auto Z_eff_ program [51], as shown in Figure 10. The Z_eff_ values of both PAC hydrogel dosimeters are nearly comparable to pure water for the photon energy above 300 keV. However, for lower energy, the Z_eff_ values of both hydrogel dosimeters are higher than the Z_eff_ of water, which is related to high photoelectric absorptions in the hydrogel dosimeters [52,53].

The maximum Z_eff_ is observed at 25.5 keV, which is the *K-edge* of Ag [54]. Significant absorption of low-energy X-ray in the case of using heavy elements is obtained due to the increase in the probability of photoelectric absorption, as this interaction is proportional to Z^3^ [55]. Additionally, the photoelectric interaction is dominant at low energies (kV ranges) [52].

**Figure 11:**
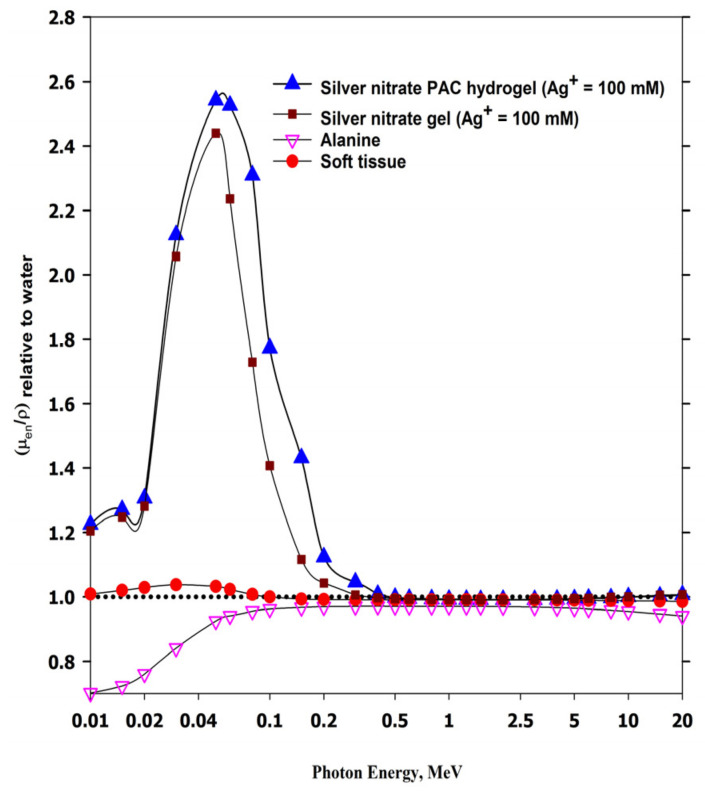
The mass energy-absorption coefficients of silver nitrate PAC hydrogel dosimeter (100 mM Ag^+^ ions) and silver nitrate gel dosimeter (100 mM Ag^+^ ions) relative to (μ_en_/ρ)_W_ of water against photon energy in the range of 0.1–20 MeV and compared with alanine dosimeter and soft tissue.

The mass-energy absorption coefficients, (μ_en_/ρ)_Hydrogel_ and (μ_en_/ρ)_Gel_ of the PAC hydrogel dosimeter and the silver nitrate gel dosimeter previously prepared [36], respectively, relative to the same values of water (μ_en_/ρ)_W_, are plotted in Figure 11 as a function of photon energy from 1 keV to 20 MeV. The mass-energy absorption coefficients were derived from the online NIST physical reference data [56]. Both dosimeters (gel and PAC hydrogel) are considered water equivalent materials in the photon energy range of 400 keV–20 MeV, exhibiting their ability to be used in radiation therapy of high-energy photons without using corrections to energy dependency.

### 2.7. Uncertainty Assessments

Various parameters can contribute to the uncertainty of absorbed dose measurements, such as calibration of the gamma irradiation cell, uniformity of silver nitrate gel dosimeter (batch homogeneity), stability of dose–response, absorbance measurement, and calibration curve fit. Table 5 lists the uncertainty parameters of the formulated gel dosimeter. The uncertainty components were determined as previously described in detail [49,57,58,59]. The response’s overall uncertainty (2σ, 95% confidence interval) was 4.04%.

### 2.8. Comparing the Response of PAC Hydrogel Dosimeter with Silver Nitrate Gel Dosimeter

**Figure 12:**
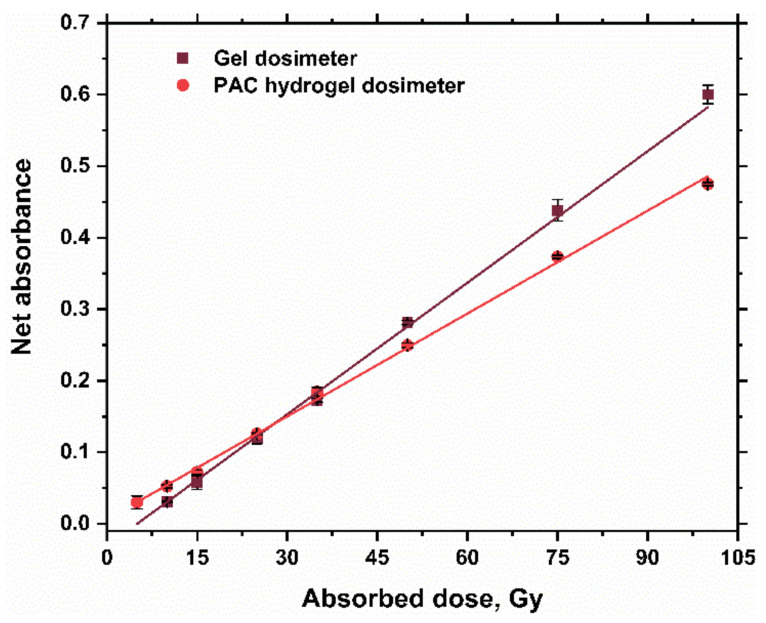
Response curves of gel dosimeter (100 mM Ag^+^ ions) at 450 nm [36] and PAC hydrogel dosimeter (100 mM Ag^+^ ions) at 453 nm.

Figure 12 displays the dose–response curves of silver nitrate PAC hydrogel and previously prepared silver nitrate gel [36] dosimeters. It was found that the response or radiation sensitivity of the gel dosimeter is higher than the PAC hydrogel, which indicates the role of gelatin in the reduction of Ag^+^ ions into Ag^o^ metallic NPs and the effect of capping agents on the response of silver nitrate dosimeter solution. However, for the low doses (less than 15 Gy), the change of response of the hydrogel is more significant than the change of response of the gel dosimeter, as shown in Figure 12. These results may indicate the effectiveness of the hydrogel dosimeter within the radio-therapeutic dose range.

## 3. Conclusions and Remarks

A novel polyacrylamide (PAC) hydrogel dosimeter based on silver nitrate was studied spectrophotometrically in a dose range of 0–100 Gy. Gamma radiation promotes the formation of Ag nanoparticles linearly up to 100 Gy, with R^2^ values of 0.9996, 0.9987, 0.9995, and 0.9986 for silver nitrate concentrations of 20, 50, 100, and 150 mM, respectively.

The responses of the PAC hydrogel dosimeters are greatly enhanced by the increase in Ag^+^ ion concentration and glycerol content. This dosimeter has a temperature coefficient of 1.6% per °C.

The Z_eff_ values of these dosimeters are comparable to the Z_eff_ values of water at energies greater than 300 keV. It was found that the PAC hydrogel dosimeter can be considered as a water equivalent material in the photon energy range of 400 keV–20 MeV.

At doses less than 15 Gy, the PAC hydrogel dosimeter exhibits higher radiation sensitivity than the gel dosimeter [36]. The overall uncertainty (2σ) in absorbed dose estimation is approximately 4.04%. These results may support a promising use of this system as a valid dosimeter to quantify the dose both in radiotherapy treatments and in blood irradiation.

## 4. Materials and Methods

### 4.1. Sample Preparation

Silver nitrate (99.8%), glycerol (99.5%), polyacrylamide (PAC), with an average molecular weight of about 5.0 × 10^5^ Da were all purchased from Aldrich, Baden-Württemberg Germany.

Aqueous PAC solutions (2% *w*/*v*) were prepared and homogenized using a magnetic stirrer at room temperature (≈ 25 °C). These solutions were then mixed with aqueous AgNO_3_ solutions to obtain dosimeter solutions with concentrations of 20, 50, 100, and 150 mM of AgNO_3_. After that, the solutions were kept under stirring for an additional hour to obtain homogeneous mixtures. The solutions were then poured slowly into disposable poly(methyl-methacrylate) (PMMA cuvettes (1 × 1 × 3 cm^3^) and stored in a refrigerator adjusted at 6 °C to form the PAC hydrogels. In addition, the effect of glycerol on the PAC response was investigated by incorporating different glycerol content of 0, 5, 15, and 25% (*v*/*v*) into PAC solutions (2% *w*/*v*) containing 100 mM of AgNO_3_. These compositions were prepared at room temperature, similar to the above-mentioned procedures. All formulations of PAC hydrogel dosimeters are shown in Table 6.

### 4.2. Samples Irradiation and Characterization

A ^60^Co Gamma Cell (GC) model GC-220 Excel (MDS, Nordion, Canada) was used to irradiate PAC hydrogel dosimeters with absorbed doses of water ranging from 0 Gy to 100 Gy. A specially designed polystyrene holder was utilized to achieve an electronic equilibrium during irradiation. The dose rate as determined by the National Physical Laboratory (NPL) in England was ≈ 1.0 kGy/h. It was measured using alanine dosimeters of the NPL (dose rate is traceable to NPL, a primary laboratory).

The γ-rays unexposed and exposed silver nitrate hydrogel dosimeters were analyzed using a UV-Vis spectrophotometer model Evolution 500 (Thermo Electron Corporation, Winsford, UK.) to various absorbed doses. This spectrophotometer was used to measure the absorptions spectra in the wavelength interval of 350–750 nm with steps of 2 nm. The optical absorbance at a fixed wavelength of 453 nm was chosen to evaluate the optical dose–response of PAC hydrogel dosimeters. Three dosimeters of each set were irradiated for each dose value.

The hydrogel responses are established with optical absorbance variation (net absorbance), ∆A = A_i_ − A_0_, where A_i_ and A_0_ are the absorbances at 453 nm for the unirradiated and irradiated hydrogel dosimeter, respectively.

## Figures and Tables

**Table 1:** Scanned images of silver nitrate hydrogel dosimeter (4% gelatin and 100 mM Ag) irradiated at different dose levels. An Epson Perfection V850 Pro scanner, made by Seiko Epson Corporation, was used to scan these images.

Absorbed Dose (Gy)	Unit
0.0	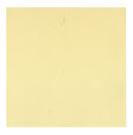
5.0	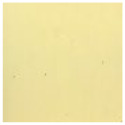
15.0	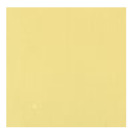
25.0	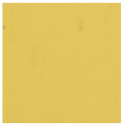
50.0	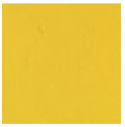
75.0	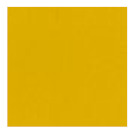
100.0	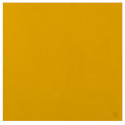

**Table 2:** Radiation sensitivity, correlation coefficient (R^2^), and the percentage variation of response of PAC hydrogel dosimeters prepared using different concentrations of silver nitrate.

Composition of PAC Hydrogel Dosimeter			Sensitivity ± S.D., Gy^−1^cm^−1^	R^2^	∆ (%)
PAC, %	[AgNO_3_], mM	Glycerol, %			
2	20	-	0.00283 ± 0.00003	0.9992	0.00
2	50	-	0.00360 ± 0.00007	0.9975	+27.21
2	100	-	0.00479 ± 0.00007	0.9987	+69.26
2	150	-	0.00427 ± 0.00009	0.9973	+50.88

**Table 3:** Radiation sensitivity, correlation coefficient, and the percentage variation of response of PAC hydrogel dosimeters prepared using different glycerol contents.

Composition of PAC Hydrogel Dosimeter			Sensitivity ± S.D., Gy^−1^cm^−1^	R^2^	∆ (%)
PAC, %	[AgNO_3_], mM	Glycerol, %			
2	100	0	0.00479 ± 0.00007	0.9987	0
2	100	5	0.00563 ± 0.00004	0.9997	+17.54
2	100	15	0.00620 ± 0.00008	0.9991	+29.43
2	100	25	0.00613 ± 0.00002	0.9968	+27.97

**Table 4:** The silver nitrate (100 mM) PAC hydrogel composition without and with 15% glycerol.

Symbol	Z Number	Fraction by Weight (without Glycerol)	Fraction by Weight (with 15% Glycerol)
H	1	0.109178	0.104578
C	6	0.010139	0.084087
N	7	0.005341	0.005341
O	8	0.864560	0.795212
Ag	47	0.010782	0.010782

**Table 5:** Uncertainty budget of PAC hydrogel dosimeter in the dose range up to 100 Gy.

Source of Uncertainty	Type of Uncertainty	Standard Uncertainty (%)
Calibration irradiation dose rate	B	1.145 ^a^
Irradiation facility	B	0.44
Instrumental variation	A	0.04
Reproducibility of measurements	A	0.42
Batch variability	A	1.03
Calibration curve fit	A	1.1
Post-irradiation stability	A	0.36
Combined standard uncertainty (u_c_), 1σ		2.02
overall uncertainty (2σ)		4.04

^a^ As quoted from the calibration certificate

**Table 6:** Different compositions of PAC hydrogel dosimeter.

PAC Hydrogel Dosimeter Composition	[PAC] % *w*/*v*	[AgNO_3_] mM	[Glycerol] % *v*/*v*
1	2	20	0
2	2	50	0
3	2	100	0
4	2	150	0
5	2	100	5
6	2	100	15
7	2	100	25

## Data Availability

Not applicable.
